# A Clinical Infrared Video-Oculoscopy Suppression Head Impulse (IR-cSHIMP) Test

**DOI:** 10.3390/audiolres14010013

**Published:** 2024-01-31

**Authors:** Vincenzo Marcelli, Beatrice Giannoni

**Affiliations:** 1 Neuro-Otology Unit, Ospedale del Mare, 80147 Naples, Italy; 2 Unit of Audiology, Department of Neuroscience, Psychology, Drug’s Area, and Child’s Health, University of Florence, 50134 Florence, Italy; beatrice.giannoni@unifi.it

**Keywords:** c-HIT, v-HIT, v-SHIMP, IR-cSHIMP, neuro-otology

## Abstract

Background: We propose a Suppression Head IMPulse (SHIMP) test method that provides for equipment only through the use of InfraRed Video-OculoScopy (IR-VOS) and allows horizontal and vertical semicircular canal function evaluation in bedside mode. We therefore named the test InfraRed clinical SHIMP (IR-cSHIMP). Methods: To check IR-cSHIMP diagnostic efficiency, we studied 22 normal subjects, 18 patients with unilateral, and 6 with bilateral deficient vestibulopathy. Each subject first underwent a vestibular examination and, only later, an IRc-SHIMP test. Results: When the IR-cSHIMP test was performed in the horizontal plane, all normal subjects showed anti-compensatory saccades. When the vertical semicircular canal function was evaluated, the same result was obtained in all normal subjects except three, which were considered false positives. In patients with vestibular deficits, the test performed in the horizontal and vertical planes were always pathological, with 100% agreement between clinical and instrumental tests. Conclusions: Our bedside method proved to be fast, simple, and effective in discriminating between healthy and pathological subjects. It required only the same skill as the better-known cHIT. For these reasons, we believe that the IR-cSHIMP should be part of daily clinical practice as a useful tool in the selection of patients to undergo more sophisticated investigations.

## 1. Introduction

Vestibular pathologies that alter the function of VOR in the different planes globally or partially, mono- or bilaterally, are common, and they require a correct and often rapid framing to establish the correct site diagnosis and therapy to ensure the patient the best course of recovery and/or compensation.

Halmagyi and Curthoys in 1988 [1] revolutionized vestibular diagnostics by introducing the clinical Head Impulse Test (cHIT), a bedside method for the qualitative assessment of dynamic horizontal VOR gain based on the detection of ocular responses to rapid head rotations in the horizontal plane. By applying a high-speed (more than 150°/s) rotational stimulus of about 15° in about 100 ms to the head of a subject staring at an earth-fixed target, a properly functioning VOR will ensure the slow compensatory eye movement in the opposite direction of head rotation sufficient to keep the target fixed on the retina.

On the contrary, in the case of a severely deficient VOR, as occurs in patients with severe peripheral vestibular hypofunction, the velocity of the VOR-generated eye movement will be insufficient to compensate for the head rotation, and the eyes will initially move together with the head. As a result of the induced retinal slip, the patient will make a compensatory refixation saccade to return gaze to the earth-fixed target.

If the reduction in horizontal VOR gain is sufficiently pronounced (at least 50%) [2] and/or the underlying pathology is acute, the examiner will be able to detect the compensatory saccade (c-saccade) with relative confidence with the naked eye.

The cHIT is a simple test to administer, requiring no special instrumentation, and for these reasons, it can be used with minimal training from the earliest stages of patient assessment. In fact, its simplicity allows its use and rough interpretation by non-specialists, even in emergency rooms. Even later, in higher-level outpatient clinics, the cHIT remains an indispensable tool in the management of the vestibulopathic patient, along with the other VOR function tests.

Unfortunately, this important vestibular test has some limitations. Besides the fact that the ability to identify c-saccades is burdened by subjectivity, one of the most important limitations is that cHIT does not pick up compensatory refixation saccades that may develop during head movements and may occur in cases of mild VOR gain deficits and in more advanced stages of pathology. Another limiting aspect of cHIT is that, although it can be performed in the plane of the vertical canals, the visualization of c-saccades in these planes, and thus the interpretation of any abnormality, is very difficult, even in the case of a significant change in the vertical VOR gain. This is due to the limited possibility of vertical excursion of the eyeballs in the orbit and the increased difficulty of performing head rotations in the vertical plane with sufficient speed and amplitude**.**

In addition, the cHIT is a clinical test and does not record eye movements. Therefore, it cannot provide a quantitative measure of VOR function and its monitoring.

To overcome the limitations of a test that is still conceptually unique, an instrumental technology has been developed over the years that allows its ocular and videographic recording, the Video Head Impulse Test (vHIT) [3], which is currently one of the most important tests for the diagnosis of vestibular disorders [4,5,6,7,8,9,10]. The vHIT uses a high-speed head-mounted camera on goggles equipped with head velocity sensors (or a remote camera) and software to measure head and eye velocities.

This method effectively quantifies and monitors VOR gain for each individual canal and provides a detailed graphic and video plotting display that is relatively quick and easy to capture and interpret.

In addition, the ability to measure the speed of eye movement also allows us to record those c-saccades that are defined as “covert” because they are generated during head movement and are therefore partially “hidden” from direct highlighting.

The ability to detect covert saccades is important not only for the effective evaluation of VOR functionality but also for monitoring the temporal evolution of a vestibulopathy and its compensation. In fact, the appearance of this type of saccade in parallel with the reduction and subsequent disappearance of overt saccades testifies to the onset of a favorable evolution of the pathology.

Thus, unlike cHIT, the diagnostic power of vHIT is not limited either by the time phase in which the patient is assessed or by the extent of receptor damage.

The introduction of instrumentation for vHIT has also allowed the development of another method for assessing dynamic VOR gain and visuo–vestibular interactions, the so-called Video Suppression Head IMPulse (vSHIMP) test. This test requires the subject to fixate a spot projected on the front wall generated by a head-mounted laser that moves synchronously in the direction of head motion [11].

In healthy subjects, during a high-frequency head impulse, a properly functioning VOR is not suppressed for the first 100 ms [12] because it is able to maintain gaze fixation in space by driving the eyes away from the head and thus away from a head-fixed target. Consequently, to regain the target at the end of the impulse, the subject must make a large, rapid eye movement in the same direction as the head rotation, an anti-compensatory saccade (ac-saccade).

In patients with reduced dynamic VOR gain, the VOR generates slow-phase eye movements that are insufficient or only partially able to move the eyes away from the head-fixed target. Depending on whether the VOR gain is reduced or absent, only small refixation saccades occur, and eventually no ac-saccades occur.

Thus, with this stimulation paradigm, the recording of ac-saccades is indicative of a normal VOR function, whereas their absence indicates a failure.

In the vSHIMP protocol, corrective ac-saccade size is also an indicator of VOR gain efficiency: healthy subjects make large saccades, and patients with vestibular loss make very small saccades or no saccades at all [13,14,15,16,17]. A recent study used vSHIMP-generated saccade size as an additional indicator of canal function. In addition, the occurrence of ac-saccades in subacute or chronic-stage patients may indicate the effectiveness of restoring dynamic VOR gain when vHIT is still pathological [18,19]. The same vHIT system provides VOR gain values and corrective saccade measurements.

As with the vHIT paradigm, the diagnostic efficacy of the vSHIMP does not depend on the stage of the vestibular pathology or its entity.

Video SHIMP has not been adapted to record eye movements in the vertical plane; therefore, with this instrumental study paradigm, we cannot currently obtain any information about the functionality of the vertical semicircular canals (VSCCs).

As proposed by D’Albora et al. [20], the SHIMP test can be performed by having the patient wear a headband that projects an LED onto the opposite wall and observing the presence/absence of corrective saccades at the end of the head impulse with the naked eye. Since this method requires direct observation of any ac-saccades, they must be large and clearly visible.

There are different types of vHIT and vSHIMP devices on the market, which differ mainly in the location of the eye movement recording camera, which in one case is independent of the patient and in the other case is integrated into a mask that also contains the head acceleration sensor. The analysis software is similar in both cases in terms of the measurements it can provide.

The vHIT and vSHIMP systems, while solving many of the problems inherent in the correct detection and quantification of eye movements resulting from high-frequency visuo–vestibular interactions, remain limited by the availability of high-tech instruments and require specific training for the acquisition of executive skills since their use is in most cases limited to neurotological specialists, at least in second- or third-level outpatient clinics.

Therefore, we wondered whether it might be possible, at least in first- and second-level vestibular outpatient settings, to overcome the need for sophisticated instrumentation while retaining the information that can be gained from studying the VOR under conditions of visual suppression.

The need to flank the cHIT with an SHIMP protocol, endowed with the same clinical practicality, led us to develop a bedside test that would require the use of only basic neuro-otological instrumentation.

To this end, we propose an extremely simple and effective method that allows us to perform a bedside SHIMP protocol for horizontal semicircular canals (HSCCs) and VSCCs testing, which we define as InfraRed clinical SHIMP (IR-cSHIMP). The only essential technology to perform this test is a standard InfraRed VideoOculoScopy (IR-VOS) system, which has been used for many years in most first/second-level neuro-otology centers for vestibular diagnostics.

In addition to the basic characteristic of being accessible to all physicians in the field, the advantage of this method could be at least threefold: (a) Being performed under IR-VOS, it allows the observation of eye movements at high magnification. This last feature is an undeniable advantage for both diagnosis and training. Actually, if the horizontal ac-saccades are indeed quite large and, often, easily perceived by the examiner, the magnification obtained with IR-VOS allows the VSCCs response, which is characterized by small ac-saccades even in cases of normality, to be easily detected without the need for more sophisticated equipment. (b) The IR-VOS equipment allows the test to be videotaped for further off-line evaluation, also by slowing down the playback speed. (c) The test is not burdened by false negatives, as there are no “covert” c-saccades.

## 2. Materials and Methods

The IR-cSHIMP requires an IR-VOS system, possibly equipped with eye movement video recording software. The system involves the use of a mask with at least one (preferably two) internal infrared video camera designed to evaluate eye movements under static and dynamic conditions.

The IR-cSHIMP requires the VOS mask to be placed on the patient’s face with a tight back strap to ensure maximum stability during active or passive head movements. For the test, it is ideal to have a monitor in front of the operator to easily evaluate the eye movement response, video recording software with slow motion playback, and an external video camera to record the examination conditions for the best online and offline interpretation of signs. When using IR-VOS, it is preferable to keep the examination environment dimly lit to improve the visibility of the images on the monitor.

The test requires a cooperative and sighted patient with no significant orthopedic problems affecting the neck.

The examiner, standing in front of or behind the patient in a seated position, holds the mask and the patient’s head firmly at the temporal level, while the subject is asked to stare carefully at the LEDs lit inside the mask throughout the examination.

Before performing the test, it is advisable to check the subject’s head mobility and relaxation of the neck muscles to explain to the subject that we are going to impose on his head a series of small, rapid, and impulsive movements in the horizontal and vertical planes and ask him to maintain maximum mobility of the cervical spine.

Once the ideal conditions for cervical mobility have been verified, the patient’s head is randomly rotated with slow trial movements of about 15° in about 100 ms (to avoid predictability) at a speed of at least 150°/s. The eye movements generated while the subject fixates on the LEDs are carefully checked on the monitor. Although it may be sufficient to perform at least two head movements (test–retest), it is preferable to perform a reasonable number of impulses to be sure of the test result.

Since our original intention was that of a bedside test, our IR-cSHIMP does not provide precise measurements of the amplitude and angular velocity of head and eye movements.

Under the test conditions described, a normal VOR responds to a rapid rotation of the head in the different planes and generates a compensatory slow eye movement in the opposite direction of the head, even when the subject is looking at the target inside the mask and therefore moving in synchrony with the head.

Once the immediate response of the VOR has been exhausted, the slow compensatory ocular slip just generated produces a retinal error that constitutes the specific stimulus for the generation of a rapid movement to re-fixate the target in an anti-compensatory direction: the ac-saccade. Such an ac-saccade is clearly visible both for rotations in the horizontal and vertical planes at the end of the head rotation, especially thanks to the magnification conditions allowed by the IR-VOS system.

On the other hand, when VOR functionality is reduced or absent, little or no slow compensatory eye slip is generated, and therefore, the eyes do not deviate from target fixation. Since there is little or no image slip on the retina, no rapid or very small anti-compensatory eye movement is detected at the end of the head rotation. When the IR-VOS is equipped with an eye tracking system, eye movements are simultaneously captured and archived. A slow-motion recording system is also useful in cases of doubtful responses.

To prove the validity of the IR-cSHIMP, we tested 22 subjects who were under observation for chronic tinnitus and never suffered from any vestibular pathology (12 women and 10 men; mean age: 43.41 years, minimum age: 24 years, and maximum age: 72 years; control group), 18 patients with unilateral vestibular deficit (8 women and 10 men; mean age: 54.55 years, minimum age: 34 years, and maximum age: 81 years; unilateral vestibular deficit—UVD group), and 6 patients with severe bilateral deficient vestibulopathy due to cerebellar ataxia with neuropathy and bilateral vestibular areflexia syndrome (CANVAS) (2 women and 4 men; mean age: 65.83 years, minimum age: 60 years, and maximum age: 75 years; bilateral vestibular deficit—BVD group).

Patients with UVD were examined in the subacute phase of the disease, selected so that they no longer had spontaneous nystagmus.

Patients with BVD also had no spontaneous signs, although they could show gaze-evoked nystagmus.

The diagnosis of unilateral and bilateral vestibulopathy was initially suspected based on general and neuro-otologic history, non-instrumental vestibular and neurologic examination, and neuro-radiologic findings.

Each subject first underwent a bedside neuro-otological examination, which was performed as follows: first, we check the integrity of the visuo-oculomotor system by grossly evaluating saccade latency, velocity, and precision, as well as the efficiency of the smooth pursuit system. Then, we check for spontaneous and gaze-evoked nystagmus, first in visual fixation conditions and then in darkness after applying the IR-VOR mask. The examination continues by looking for positional nystagmus with the patient in the supine, right and left lateral, and head hanging positions, and by performing right and left Dix-Hallpike and horizontal Pagnini-McClure positioning tests. We also perform vestibular function assessments such as head shaking, visuo–vestibular interaction tests such as VOR cancellation and visual VOR in the dark, or fixation conditions in which each test should be performed.

Finally, subjects underwent the IR-cSHIMP test, either by direct observation or by simultaneous recording of the oculomotor response.

After the IR-cSHIMP test, the subjects also underwent the vHIT and vSHIMP test protocols, using the method described in the introduction. We would like to point out that we used the ICS Impulse^®^, GN Otometrics, Taastrup, Denmark, (software: Otosuite Vestibular. 4.10 Build 1341).

The IR-cSHIMP results were reviewed separately and blinded by two experienced neuro-otologists (VM and BG), and their judgments were then compared.

Only after this preliminary double comparison were the results of the IR-cSHIMP assay compared with those of the vHIT and vSHIMP tests.

To verify the sensitivity of the clinical test performed in the horizontal plane, we compared what was seen qualitatively by the IR-cSHIMP test with what was assessed qualitatively and quantitatively by vSHIMP. With respect to the vertical plane, since vertical vSHIMP has not yet been developed, we compared what was seen qualitatively with the IR-cSHIMP test to what was assessed quantitatively with vHIT.

The study was conducted in accordance with the tenets of the Declaration of Helsinki.

Subjects were not specifically recruited for the study but attended our department as outpatients. Healthy subjects underwent vestibular examinations because they suffered from tinnitus.

At the time of data processing, neither the internal control of our hospital nor the ethics committee deemed approval necessary.

## 3. Results

### 3.1. Normal Subjects, n = 22

#### 3.1.1. Bedside Examination

All 22 subjects belonging to this group did not complain of any vestibular disturbance. However, they were subjected to a vestibular examination as part of a complete neuro-otological evaluation carried out because they were experiencing tinnitus.

All examined subjects showed normal ocular motricity, had normal saccades for all examined parameters, and normal bedside smooth pursuit testing. Spontaneous, positional, and positioning nystagmus were absent in all cases, and VOR function tests were negative for hypofunction or asymmetries.

#### 3.1.2. Instrumental Tests

**Figure 1:**
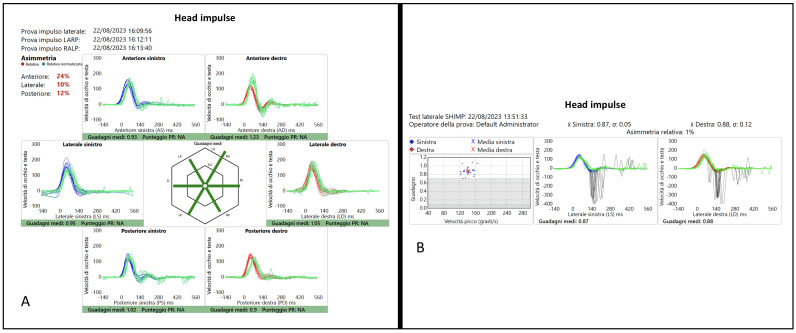
(**A**) Horizontal and vertical vHIT and (**B**) vSHIMP test plotting in a control subject (n. 6—MA).

When IR-cSHIMP was performed in the horizontal plane, all the control subjects showed clear ac-saccades in both directions. An example (subject six, MA) is reported in Figure 1 and Video S1.

By performing IR-cSHIMP in the vertical plane, considering that 22 subjects and therefore 44 VSCCs were examined, we also observed ac-saccades in all but 3 cases (41/44 VSCCs—93.18%). In the latter three cases (6.82%; pts. 1, 5, and 12), ac-saccades were absent even when the slow-motion video recordings were analyzed (Table 1).

When studying the VSCC function, the agreement between instrumental methods and the IR-cSHIMP test can only be assessed by comparing its results with those of the vHIT. Therefore, in control subjects, the agreement occurred in 93.18% of cases.

### 3.2. Patients with Unilateral Vestibular Deficit, n = 18

#### 3.2.1. Bedside Examination

The 18 patients belonging to this group were affected by a unilateral vestibular hypofunction attributable in 13 cases to a vestibular neuritis, in 4 subjects to a peripheral vestibulopathy on a vascular basis, and in 1 patient to a vestibular Schwannoma. In any case, they all had been recruited in at least a subacute phase of the disease, when they no longer presented spontaneous nystagmus. Saccades and smooth pursuit of bedside testing were normal in all cases, showing neither intrinsic or extrinsic alterations. None of the patients showed gaze-evoked nystagmus nor vertical or horizontal positioning nystagmus. A horizontal/torsional positional nystagmus beating towards the unaffected side was still present in 12 out of 18 patients. The cHIT was positive, showing a clear c-saccade in all patients, and the head shaking test revealed, in all UVD subjects, a nystagmus beating towards the healthy side.

#### 3.2.2. Instrumental Tests

Six of the eighteen patients had only an HSCC VOR gain deficit when they were tested with vHIT/vSHIMP. Of these six subjects, two (Pts 4 and 6) had a normal IR-cSHIMP showing ac-saccades even when the head is turned to the injured side. The remaining four showed an abnormal IR-cSHIMP test with no ac-saccade when turning to the lesioned ear. However, the IR-cSHIMP test results of all six patients were exactly reproduced by vSHIMP.

Twelve of the eighteen patients showed both horizontal and vertical vHIT-documented unilateral SCCs VOR gain deficits. Of these, the IR-cSHIMP test was normal in only 1/12 patients (Pt 15). In fact, in 9/12 patients (Pts 7–14 and 18) the test showed an altered anti-compensatory response for both horizontal and vertical SCC testing; in 1/12 patients (Pt 16) an altered anti-compensatory response only for HSCC stimulation; and in 1/12 patients (Pt 17), an altered anti-compensatory response only for VSCC stimulation.

**Figure 2:**
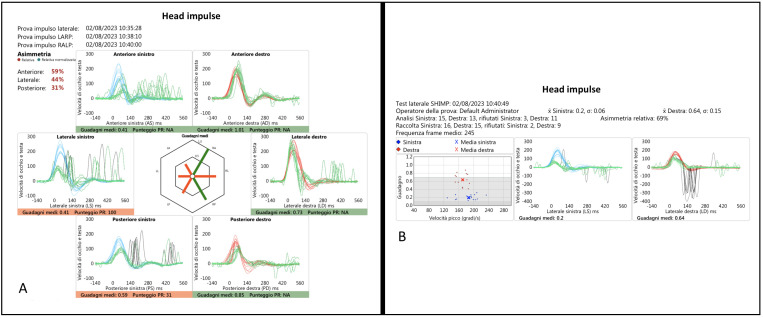
(**A**) vHIT plot showing a left horizontal and vertical SCC VOR gain deficit and a slight “compensatory” right HSC VOR gain reduction; (**B**) vSHIMP traces showing the absence of ac-saccades with head rotation to the left.

The results of horizontal and vertical vHIT of a patient (Pt 11, SG) illustrating a left vestibulopathy and a very slight right deficit, probably compensatory in nature, are shown in Figure 2, and the corresponding videotaped IR-cSHIMP test is shown in the linked Video S2.

In three of the patients with a pathological horizontal IR-cSHIMP test (Pts 10, and 14, 16), the ac-saccades were small but still well detectable and confirmed by vHIT/vSHIMP.

**Figure 3:**
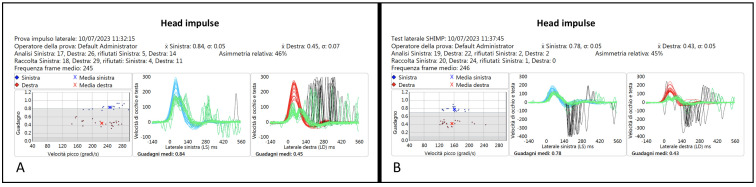
(**A**) vHIT traces of a patient with a right HSCC VOR gain deficit; differently, the vSHIMP test (**B**) demonstrates the presence of anti-compensatory saccades by rotating the head towards the right, only slightly reduced in amplitude with respect to the contralateral.

The vHIT and vSHIMP traces of one of these three similar patients (Pt 16, CF) are shown in Figure 3, and the corresponding videotaped IR-cSHIMP test is reported in the linked Video S3.

In four doubtful cases regarding VSCCs IR-cSHIMP test results, we successfully resolved the uncertainty by using slow motion analysis, which confirmed the absence of anti-compensatory saccades in this plane.

Horizontal SCCs IR-cSHIMP results were always confirmed by horizontal vSHIMP, with the agreement between the two methods being 100%.

Since there is no vSHIMP method to test the functionality of the VSCCs, the vertical IR-cSHIMP test results were only comparable to those of the vertical vHIT. However, even in these cases, the IR-cSHIMP test was indicative of what would be expected based on the results of the instrumental examination (Table 2).

### 3.3. Patients with Bilateral Vestibular Deficit, n = 6

#### 3.3.1. Bedside Examination

All six patients in this group had BVD as a sign of CANVAS syndrome. At the time of the vestibular bedside examination, saccade accuracy and velocity were still normal. On the contrary, the smooth pursuit eye movements were restructured, being burdened by catch-up saccades in both horizontal and vertical planes. None of the patients showed peripheral or central spontaneous–positional signs, but all of them had a gaze-evoked down-beating nystagmus, detectable both under fixation and in its absence. In all patients, the horizontal cHIT was positive for a bilateral corrective c-saccade, while the vertical cHIT was not correctly executed and reliable (also because of gaze-evoked down-beating nystagmus).

The visual VOR was pathologically enhanced in all subjects, and the VOR cancellation test showed a lack of reflex inhibition in three out of six cases.

The head shake test showed down-beating post-head shaking nystagmus in four out of six patients of the group.

#### 3.3.2. Instrumental Tests

**Figure 4:**
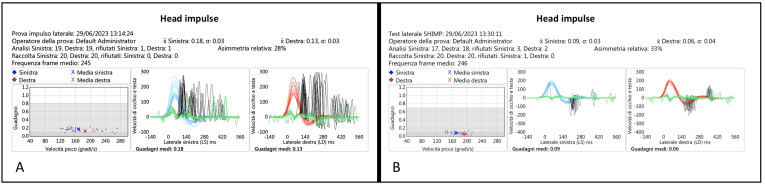
(**A**) vHIT plotting of patient 4 (CS), with a bilateral HSCCs VOR gain deficit; differently, in this case, the vSHIMP test (**B**) demonstrates the presence of small anti-compensatory saccades by rotating the head in both directions.

All patients in this group (Table 3) had a bilateral horizontal and vertical vHIT-documented SCC VOR gain deficit ranging from moderate to severe. In all but one patient, the IR-cSHIMP also showed the absence of ac-saccades in both planes. In the patient who was the exception (Pt 4, CS), some ac-saccades were still visible when the test was performed in the horizontal plane, although they were of very small amplitude. In any case, such a peculiar characteristic of the response was also confirmed by vSHIMP (Figure 4 and Video S4).

Considering the results, however, even in the case of a bilateral deficient vestibulopathy, the percentage of agreement between instrumental tests (vSHIMP for HSCCs and vHIT for VSCCs) and IR-cSHIMP was 100%.

No subjects reported any adverse effects due to the administration of the test.

## 4. Discussion

In daily neuro-otological practice, in first- and second-level outpatient clinics and in the emergency room, the need to assess visual-vestibular interactions to determine the possible presence of primary or secondary, mono- or bilateral, VOR hypofunction is common, both for diagnostic and differential diagnostic purposes. At the same time, it is also important to establish the timing of the development of the pathology and, thus, its prognosis.

In such contexts, at least in Italy, it is uncommon to always arrange sophisticated and still expensive instruments such as the vHIT/vSHIMP, which would allow the acquisition of pathological signs and their quantification. In fact, the equipment available in these situations is most often simpler, with the eye movement capture instrument consisting of IR-VOS alone, often coupled only with a system for recording and archiving video images.

On the other hand, the non-instrumental or basic-instrumental vestibular examination still retains a role of fundamental diagnostic importance, certainly not being fully replaced but rather completed and detailed by third-level instrumental tests like vHIT/vSHIMP or instrumental otolith function testing.

For the bedside neuro-otological assessment, for many years we have had the cHIT available, a test that, albeit with some limitations, allows us a rapid and often valid qualitative assessment of the horizontal dynamic VOR gain in the absence of visual suppression.

To date, an equally reliable and practical system for studying the VOR functionality in visual suppression conditions has not been proposed, while it would be of great absolute and contextual value as well as being important in the monitoring of vestibulopathy. In essence, what has been missing up to now is a clinical SHIMP test that does not require too sophisticated instrumentation and is therefore available to all specialists, including first- and second-level approaches to the patient.

D’Albora et al. [20] recently proposed a SHIMP test that can be performed by having the patient wear a headband that projects an LED onto the wall and observing, with the naked eye, the presence/absence of any corrective ac-saccades at the end of the head impulses. Even this test, although it does not absolutely require a machine to record and measure eye movements, still requires the use of a special mask to generate an external LED, which is therefore an apparatus not commonly available. Moreover, in such a test, the detection of the eye movements induced by the rapid rotation in the state of visual suppression is entrusted only to the direct control of the examiner and then conditioned by the amplitude of the movements themselves.

From vHIT/vSHIMP practice, we know that horizontal corrective saccades are often wide enough and therefore relatively easy to detect, but we also experience that those in the vertical plane are more difficult to detect due to problems with vertical eye movements in the orbit and the lower gain of the VOR in this plane. For these reasons, we believe that the test proposed by Albora [20], although conceptually flawless, cannot be considered a practical clinical alternative to vSHIMP.

Therefore, we developed and tested a clinical SHIMP method to be placed alongside other bedside tests routinely used to assess vestibular function, designed to be very simple, quick to perform and interpret, and capable of providing sufficiently safe and repeatable results. We also wanted the test to be widely available to operators in the field. We named the test “IR-cSHIMP” because it represents the clinical variant of the analogue instrumental vSHIMP and because it is performed using only the IR-VOS, a basic piece of equipment now owned and used in all second- and even first-class neuro-otology clinics.

Like vSHIMP, the IR-cSHIMP test is designed to evaluate oculomotor responses to rapid head rotations under conditions of fixation of a visual target that is integral to the head. In the IR-cSHIMP test, the target is simply the illuminated LED inside the standard IR-VOS mask. To detect eye movements under these conditions, it is sufficient for the examiner to be accustomed to doing so as in a normal basic neuro-otological examination.

The association of a video recording system with the IR-VOS increases the sensitivity and efficiency of the IR-cSHIMP test by allowing oculomotor findings to be archived and analyzed offline, even at reduced playback speeds. In the sample we studied, such a tool was fundamental in resolving the interpretive doubts about the actual absence of even small vertical saccades in four patients with unilateral deficient vestibulopathy.

Because the images of eye movements during the test can be viewed directly on the monitor by several people at the same time, the IR-cSHIMP test can also be used for clinical comparison and student teaching.

Moreover, from the moment that the impulsive movements to be conveyed to the patient’s head during the IR-cSHIMP test are the same as those normally used in performing cHIT, it is not necessary for the examiner to have a special training period since this should be a posed skill.

Regarding the patient who can be subjected to the IR-cSHIMP test, we found it necessary only that he/she be a cooperative subject, sighted, able to sit up, and without any pathological condition that could reduce the mobility of the cervical spine or cause neck pain. Once these conditions were checked, the test proved to be non-invasive, easy to accept, and easy to understand for all the subjects studied.

Compared to cHIT and vHIT, we found a greater tendency for subjects to be distracted from looking at the visual target when performing the IR-cSHIMP test. We believe that this tendency is due to a greater difficulty in identifying the target in a dark context compared to the environmental contextualization condition, as occurs in the cHIT/vHIT. By frequently reminding the subject not to lose the target, this disadvantage was easily overcome.

To avoid any conditioning, the IR-cSHIMP result was evaluated separately and at different times by two expert neuro-otologists so that the second assessment was considered a re-test to check the reproducibility of the results.

The intra-examiner repeatability of the IR-cSHIMP test was total. In fact, even limiting the number of cephalic movements, the examiner’s individual judgment of the test result was always test/retest concordant.

The blinded comparison of the IR-cSHIMP test results of the two different examiners also showed 100% agreement. This means that the test had no room for subjective interpretation and that the results are repeatable, as the assessment of the same subject was carried out by the two examiners at different times. Inter-individual agreement was complete even in cases where the test result was considered doubtful in the baseline conditions, and it was later necessary to resort to the analysis of eye movements in slow motion.

In our study, we made sure to test the IR-cSHIMP method on two categories of people: normal subjects and dizzy patients.

Normal individuals were subjects who were yet to undergo a vestibular examination because they complained of tinnitus. Furthermore, these subjects had never reported any vertiginous symptoms, nor was there any documented vestibular pathology in their history. These requirements ensured that the basic functionality of the VOR was considered within normal parameters.

To evaluate the efficiency and reliability of the IR-cSHIMP test in pathological conditions, we also examined two groups of homogeneous patients with established, non-hydropic, unilateral or bilateral peripheral vestibulopathy. Such vestibular pathologies usually show a clear abnormality of the recorded ac-saccades and a regular progression over time. In addition, unilateral or bilateral vestibulopathy is a condition in which testing the visually suppressed VOR function provides the most useful diagnostic contribution.

The three groups of subjects were found to be homogeneous and representative of the conditions of normal vestibular function and unilateral and bilateral vestibular hypofunction based on the results of bedside and instrumental vestibular examinations. For this reason, we believe that the validation of the IR-cSHIMP test on these subjects can be considered effective and descriptive of the conditions tested.

Patients were also selected so that they no longer showed spontaneous nystagmus because such a biphasic eye movement could have interfered by altering the results. On the other hand, we believe that if the IR-cSHIMP test is to enter clinical routine, it should also be tested under these conditions to evaluate the possible relationships between nystagmus direction and angular velocity and the presence/absence and amplitude of ac-saccades.

The IR-cSHIMP test has been shown to be able to assess both horizontal and vertical SCC function by directly detecting the presence/absence of ac-saccades generated under specific conditions.

In the study of HSCC function, agreement between clinical and instrumental examinations was verified by comparing eye movements detected during the IR-cSHIMP test with both the vHIT trace and the corresponding vSHIMP. With the vHIT, we were able to calculate VOR gain values and verify the presence/absence and amplitude of overt and/or covert c-saccades; with the horizontal vSHIMP test, we were able to record the presence/absence and amplitude of ac-saccades.

With this type of assessment, both in normal subjects and in patients with unilateral or bilateral deficient vestibulopathy, the agreement between the IR-cSHIMP test and instrumental test outcomes was complete.

We also verified a close correlation between the amplitude of horizontal ac-saccades, as detected by IR-cSHIMP, when present, and the VOR gain values estimated by vHIT: the greater the amplitude of the anti-compensatory rapid eye movements, the more valid the VOR gain.

When we tested the function of the vertical canals, we could only compare IR-cSHIMP results with those of vertical vHIT. Also, in this case, however, vHIT enabled the calculation of right anterior-left posterior and left anterior-right posterior VOR gains and the verification of the presence/absence and amplitude of c-saccades. Therefore, it was possible to determine whether the perception of adequate ac-saccades at the vertical IR-cSHIMP test corresponded to normal vertical VOR gains or, conversely, whether they were absent or of low amplitude in the case of reduced vertical VOR gains.

In assessing VSCC function, we paradoxically noted that the congruence between clinical and instrumental methods was lower when evaluating healthy subjects with respect to patients, for which the correspondence of the data was instead complete. Among normal individuals, the vertical IR-cSHIMP test was pathological (i.e., without any ac-saccade) in 3/44 (6.82%) of the VSCCs whose vHIT documented gain was, obviously, normal. In such cases, not even the slow-motion examination was able to detect any corrective rapid movement. The possibility of having false positives at the IR-cSHIMP test when assessing VSCC function must certainly be considered. However, it is more acceptable to have to investigate further in a subject who will later turn out to be healthy than the contrary, that is, to lose a pathological subject with a falsely negative test. The latter situation has never occurred in our samples, neither by studying the horizontal nor the vertical SCCs.

Anyway, as it happens for vHIT, the detection of ac-saccades during vertical IR-cSHIMP was also less immediate, both due to the greater difficulty in stimulating VSCCs and due to the reduced possibility of vertical eye excursion. This difficulty represents a limitation of the test, although, facing a peripheral vestibular deficit, it must be noted that the IR-cSHIMP error rate was zero and that slow motion video recording has been successfully used in cases of particularly difficult interpretation.

In addition, operator experience, comparison with the contralateral response, and association with other pathologic signs will certainly help reduce the error rate.

Among patients with subacute unilateral vestibulopathy manifesting a horizontal vHIT-documented SCC VOR gain deficit, we had four (Pts 4, 6, 15, and 17) in whom the IR-cSHIMP, however, showed some ac-saccades when turning the head to the hypofunctioning side. In practice, the only apparent inconsistency between vHIT reports and IR-cSHIMP test results was seen in these four patients.

On the other hand, the same discrepancy was also found between vHIT and vSHIMP results, with the latter recording the same anti-compensatory saccades as detected with the IR-cSHIMP test.

Therefore, our data confirm the congruence between IR-cSHIMP and vSHIMP, even in the case of certain patients, and confirm the efficiency of IR-cSHIMP, as well as vSHIMP, in detecting the tendency of ac-saccades to reappear earlier than compensatory ones in a favorable evolution of a UVD [18,19].

Among the patients who, despite a complete canal deficit, showed some anti-compensatory response, there were also three in whom saccades of very small dimensions were detected on both the IR-cSHIMP and the horizontal vSHIMP tests. Even in these subjects, the IR-cSHIMP was as sensitive as the vSHIMP in detecting the tendency to early recovery of this visuo–vestibulo–oculomotor function.

In conclusion, we believe that the IR-cSHIMP test has proven to be extremely useful and simple enough to be performed by all practitioners who normally deal with patients with vestibular disorders, even in basic outpatient or emergency department settings. IR-cSHIMP has also shown a very high sensitivity and specificity in discriminating between normal and pathological subjects, therefore responding to the purposes for which it was designed.

The IR-cSHIMP test is very rapid; therefore, it can easily be added to the routine vestibular examination without adding too much time, but rather provides important diagnostic information about visual-vestibular interactions that are otherwise not always assessed during bedside vestibular testing.

As with other eye movements studied in the vestibular examination, the possibility of recording those generated during IR-cSHIMP allows their qualitative monitoring during the temporal evolution, both of a previous normal state and of pathology.

Although we believe that the number of subjects we have studied is sufficient for the evaluation of the first results of the proposed test, it is certainly desirable to increase its application to both normal and pathological subjects. Among the latter, it will be appropriate in the future to consider both subjects with other types of pathologies, for example, hydropic conditions, and patients with spontaneous nystagmus, to evaluate the influence of the latter sign on the results.

## 5. Conclusions

The IR-cSHIMP test we propose has proven to be extremely useful and simple enough to be performed by all practitioners who normally deal with patients with vestibular disorders, even in basic outpatient or emergency department settings. It has shown very high sensitivity and specificity in discriminating between normal and pathological subjects.

The IR-cSHIMP test is very fast, so it can be easily added to the routine vestibular examination without adding time expenditure, rather than providing important diagnostic information on visuo–vestibular interactions otherwise not investigated during bedside vestibular testing.

The possibility of recording eye movements during the IR-cSHIMP allows their qualitative monitoring during the temporal evolution of both a previous normal state and pathology.

Although we believe that the number of subjects we have examined is sufficient for the evaluation of IR-cSHIMP preliminary results, it is certainly desirable to increase its application to both normal and pathological subjects. Among the latter, it will be appropriate in the future to consider both subjects with other types of pathologies and patients with spontaneous nystagmus, the latter to assess the influence of the sign on results.

The IR-cSHIMP test should in no way be considered a substitute for instrumental testing, but we believe it could be a useful diagnostic tool for selecting those patients who should instead be sent for more in-depth vestibular testing.

## Figures and Tables

**Table 1:** Normal subjects instrumental test results.

	Normal Subjects	Horizontal SCC vHIT Gain		Horizontal SCC vSHIMP Gain		Horizontal SCC IRc-SHIMP ac-S		Vertical SCC vHIT Gain				Vertical SCC IRc-SHIMP ac-S	
	ID	Left	Right	Left	Right	Right	Left	LA	RP	RA	LP	Impulse up	Impulse down
1	CL, 39 yo, f	0.84	0.87	0.80	0.81	Present	Present	0.93	0.90	1.23	1.02	**Not evident**	Present
2	ADS, 62 yo, f	0.91	0.88	0.83	0.87	Present	Present	0.88	0.79	1.02	1.00	Present	Present
3	RT, 45 yo, m	0.83	0.90	0.82	0.88	Present	Present	0.99	0.94	0.93	1.03	Present	Present
4	SU, 25 yo, f	0.87	0.81	0.82	0.84	Present	Present	0.93	0.90	1.23	1.02	Present	Present
5	EF, 43 yo, f	0.89	0.88	0.86	0.89	Present	Present	0.76	0.84	0.86	0.89	Present	**Not evident**
6	MA, 40 yo, m	0.95	1.05	0.87	0.88	Present	Present	0.93	0.90	1.23	1.02	Present	Present
7	RL, 30 yo, f	0.87	0.81	0.84	0.82	Present	Present	0.86	0.79	0.91	0.88	Present	Present
8	ML, 71 yo, f	1.08	1.04	1.00	1.09	Present	Present	0.96	0.91	1.12	1.03	Present	Present
9	EM, 24 yo, m	0.88	0.92	0.83	0.89	Present	Present	0.77	0.84	0.78	0.73	Present	Present
10	LS, 41 yo, f	0.83	0.88	0.81	0.84	Present	Present	0.97	0.91	1.00	0.99	Present	Present
11	EM, 50 yo, m	0.91	0.87	0.86	0.84	Present	Present	0.93	0.94	0.89	0.90	Present	Present
12	EDB, 34 yo, f	0.87	0.81	0.85	0.82	Present	Present	0.93	0.90	1.12	1.02	**Not evident**	Present
13	PL, 72 yo, m	0.84	0.92	0.83	0.87	Present	Present	0.76	0.82	0.80	0.86	Present	Present
14	BA, 65 yo, m	0.82	0.87	0.83	0.84	Present	Present	0.88	0.83	0.86	0.92	Present	Present
15	LV, 63 yo, m	0.93	0.88	0.90	0.86	Present	Present	0.97	0.94	1.01	1.02	Present	Present
16	MS, 58 yo, m	0.90	0.85	0.91	0.88	Present	Present	0.93	0.99	0.97	0.93	Present	Present
17	PA, 56 yo, f	0.83	0.81	0.83	0.85	Present	Present	0.81	0.85	0.83	0.78	Present	Present
18	FL, 70 yo, f	0.89	0.92	0.86	0.89	Present	Present	0.76	0.82	0.82	0.84	Present	Present
19	SB, 48 yo, m	0.84	0.81	0.83	0.80	Present	Present	0.75	0.81	0.77	0.84	Present	Present
20	FT, 59 yo, m	0.90	0.85	0.86	0.83	Present	Present	0.93	0.92	0.89	0.90	Present	Present
21	DP, 71 yo, f	0.92	0.88	0.84	0.83	Present	Present	0.83	0.88	0.85	0.86	Present	Present
22	SG, 60 yo, f	0.89	0.92	0.82	0.81	Present	Present	0.91	0.94	1.01	0.89	Present	Present

Legend. SCC: Semicircular Canal; ac-S: anti compensatory Saccade; LA: Left Anterior; RP: Right Posterior; RA: Right Anterior; LP: Left Posterior.

**Table 2:** Patients with unilateral vestibular deficit.

	UVD Patients	H-vHIT G		H-vSHIMP G		H-IRc-SHIMP	V-vHIT G		V- IRc-SHIMP
	ID	Side	c-S	Side	ac-S	ac-S	SCC	c-S	ac-S
1	VC, 81 yo, f	R: 0.27	O	R: 0.25	Absent	Present	Normal	Absent	Present
2	TDM, 42 yo, m	L: 0.51	O	L: 0.48	Absent	Present	Normal	Absent	Present
3	LG, 72 yo, f	L: 0.51	O	L: 0.40	Absent	Present	Normal	Absent	Present
4	SV, 38 yo, f	L: 0.44	C	L: 0.40	**Present**	**Present**	Normal	Absent	Present
5	MA, 69 yo, f	L: 0.38	O	L: 0.30	Absent	Present	Normal	Absent	Present
6	BT, 56 yo, m	R: 0.41	O/C	R: 0.32	**Present**	**Present**	Normal	Absent	Present
7	SN, 51 yo, m	R: 0.28	O	R: 0.33	Absent	Absent	A-P: 0.44; 0.39	O	Absent
8	TF, 56 yo, f	R: 0.38	O	R: 0.42	Absent	Absent	A-P: 0.28; 0.40	O	Absent
9	DB, 44 yo, f	R: 0.27	O	R: 0.29	Absent	Absent	P: 0.10	O	Absent
10	CL, 52 yo, f	L: 0.31	O/C	L: 0.37	**Present**	**Small**	A: 0.18	O	Absent
11	SG, 63 yo, m	L: 0.41	O/C	L: 0.20	Absent	Absent	A-P: 0.41; 0.59	O	Absent
12	ADC, 57 yo, m	R: 0.33	O/C	R: 0.30	Absent	Absent	P: 0.14	O	Absent
13	CR, 68 yo, m	R: 0.18	O	R: 0.12	Absent	Absent	P: 0.08	O	Absent
14	AV, 47 yo, m	R: 0.24	O	R: 0.20	**Present**	**Small**	A-P: 0.34; 0.44	O	Absent
15	FL, 34 yo, f	L: 0.31	C	L: 0.35	**Present**	**Present**	P: 0.38	O	**Present**
16	CF, 74 yo, m	R: 0.45	O/C	R: 0.43	**Present**	**Small**	P: 0.20	O	**Present**
17	LV, 63 yo, m	L: 0.57	C	L: 0.45	**Present**	**Present**	A: 0.22	O	Absent
18	SW, 37 yo, m	R: 0.13	O	R: 0.08	Absent	Absent	Present	O	Absent

Legend. c-Saccade: compensatory Saccade; ac-S: anti compensatory Saccade; SCC: Semicircular Canal; A: Anterior; P: Posterior.

**Table 3:** Patients with bilateral vestibular deficit.

	BVD Patients	Horizontal SCC Gain vHIT			Horizontal SCC Gain vSHIMP			Horizontal IRc SHIMP	Vertical SCC Gain vHIT					Vertical IRc-SHIMP
	ID	Left	Right	S	Left	Right	ac-S	ac-S	LA	LP	RA	RP	ac-S	ac-S
1	OMG,75 yo, f	0.60	0.53	O	0.49	0.50	A	A	0.33	0.33	0.49	0.29	A	Absent
2	AF, 61 yo, m	0.19	0.31	O	0.22	0.34	A	A	0.05	0.30	0.19	0.20	A/O/S	Absent
3	AG, 68 yo, m	0.45	0.38	O	0.44	0.40	A	A	0.49	0.38	0.43	0.33	O	Absent
4	CS, 61 yo, m	0.18	0.13	O/C	0.09	0.06	VS	VS	0.20	0.17	0.23	0.17	A	Absent
5	SG, 60 yo, m	0.26	0.26	O/S	0.27	0.27	A	A	0.15	0.23	0.33	0.43	A	Absent
6	UR, 70 yo, f	0.6	0.53	O	0.43	0.50	A	A	0.24	0.30	0.34	0.27	A	Absent

Legend. SCC: Semicircular Canal; S: Saccade; O: Overt; C: Covert; A: Absent; VS: Very Small; S: Small; ac-S: anti compensatory Saccade; LA: Left Anterior; RP: RP: Right Posterior; RA: Right Anterior; RP: Right Posterior.

## Data Availability

The data that supports the findings of this study are available on request from the corresponding author. The data are not publicly available due to privacy or ethical restrictions.

## Supplementary Materials

The following supporting information can be downloaded at https://www.mdpi.com/article/10.3390/audiolres14010013/s1. Video S1. Normal HSCC IR-cSHIMP; Video S2. Left HSCC deficit, abnormal vSHIMP; Video S3. Right HSCC deficit, normal IR-cSHIMP; Video S4. Bilateral HSCC Deficit, Deficit IRc-SHIMP.

## References

[1] Halmagyi G.M., Curthoys I.S. (1988). A clinical sign of canal paresis. Arch. Neurol..

[2] Barin K. (2019). Estimating loss of canal function in the video head impulse test (vHIT). J. Vestib. Res..

[3] MacDougall H.G., Weber K.P., McGarvie L.A., Halmagyi G.M., Curthoys I.S. (2009). The video head impulse test: Diagnostic accuracy in peripheral vestibulopathy. Neurology.

[4] Rambold H.A. (2015). Economic management of vertigo/dizziness disease in a county hospital: Video-head-impulse test vs. caloric irrigation. Eur. Arch. Otorhinolaryngol..

[5] Manzari L., Graziano D., Tramontano M. (2020). The Different Stages of Vestibular Neuritis from the Point of View of the Video Head Impulse Test. Audiol. Res..

[6] van Esch B.F., Nobel-Ho G.E., van Benthem P.P., van der Zaag-Loonen H.J., Bruintjes T.D. (2016). Determining vestibular hypofunction: Start with the video-head impulse test. Eur. Arch. Otorhinolaryngol..

[7] Kontorinis G., Tailor H., Tikka T., Slim M.A.M. (2023). Six-canal video head impulse test in patients with labyrinthine and retrolabyrinthine pathology: Detecting vestibulo-ocular reflex deficits. J. Laryngol. Otol..

[8] Wettstein V.G., Weber K.P., Bockisch C.J., Hegemann S.C. (2016). Compensatory saccades in head impulse testing influence the dynamic visual acuity of patients with unilateral peripheral vestibulopathy. J. Vestib. Res..

[9] Curthoys I.S., McGarvie L.A., MacDougall H.G., Burgess A.M., Halmagyi G.M., Rey-Martinez J., Dlugaiczyk J. (2023). A review of the geometrical basis and the principles underlying the use and interpretation of the video head impulse test (vHIT) in clinical vestibular testing. Front. Neurol..

[10] Faranesh N., Abo-Saleh K., Kaminer M., Shupak A. (2023). Refining the Video Head Impulse Test Diagnostic Accuracy: A Case-Control Study. Audiol. Neurootol..

[11] MacDougall H.G., McGarvie L.A., Halmagyi G.M., Rogers S.J., Manzari L., Burgess A.M., Curthoys I.S., Weber K.P. (2016). A new saccadic indicator of peripheral vestibular function based on the video head impulse test. Neurology.

[12] Crane B.T., Demer J.L. (1999). Latency of voluntary cancellation of the human vestibulo-ocular reflex during transient yaw rotation. Exp. Brain Res..

[13] Manzari L., Orejel Bustos A.S., Princi A.A., Tramontano M. (2022). Video Suppression Head Impulses and Head Impulses Paradigms in Patients with Vestibular Neuritis: A Comparative Study. Healthcare.

[14] van Dooren T., Starkov D., Lucieer F., Dobbels B., Janssen M., Guinand N., Fornos A.P., Kingma H., Van Rompaey V., van de Berg R. (2022). Suppression Head Impulse Test (SHIMP) versus Head Impulse Test (HIMP) When Diagnosing Bilateral Vestibulopathy. J. Clin. Med..

[15] Park J.S., Lee J.-Y., Nam W., Noh S., Chang S.O., Kim M.B. (2020). Comparing the Suppression Head Impulse Paradigm and the Head Impulse Paradigm in Vestibular Neuritis. Otol. Neurotol..

[16] Elsherif M. (2023). The Conventional Head Impulse Test Versus the Suppression Head Impulse Test: A Clinical Comparative Study. J. Int. Adv. Otol..

[17] Starkov D., Vermorken B., Van Dooren T.S., Van Stiphout L., Janssen M., Pleshkov M., Guinand N., Fornos A.P., Van Rompaey V., Kingma H. (2021). The Effect of Different Head Movement Paradigms on Vestibulo-Ocular Reflex Gain and Saccadic Eye Responses in the Suppression Head Impulse Test in Healthy Adult Volunteers. Front. Neurol..

[18] Shen Q., Magnani C., Sterkers O., Lamas G., Vidal P.-P., Sadoun J., Curthoys I.S., de Waele C. (2016). Saccadic velocity in the new suppression head impulse test: A new indicator of horizontal vestibular canal paresis and of vestibular compensation. Front. Neurol..

[19] Casani A.P., Canelli R., Lazzerini F., Navari E. (2021). Prognosis after acute unilateral vestibulopathy: Usefulness of the suppression head impulse paradigm (SHIMP). J. Vestib. Res..

[20] D’albora R., Noboa R., Road J.C., Gutman M.C., Fuentes S.F., Palazón C.V.A., Silveira L., Moreno C.A.F., Monaco M.J., Zalazar G.J. (2022). Clinical Testing of Head Impulse Paradigm and Suppression Head Impulse Paradigm Using a Diagnostic Headband: Combined Clinical Sign for Improved Performance. Otol. Neurotol..

